# The paradox of plant preference: The malaria vectors *Anopheles gambiae* and *Anopheles coluzzii* select suboptimal food sources for their survival and reproduction

**DOI:** 10.1002/ece3.11187

**Published:** 2024-03-25

**Authors:** Prisca S. L. Paré, Domonbabele F. D. S. Hien, Mariam Youba, Rakiswendé S. Yerbanga, Anna Cohuet, Louis‐Clément Gouagna, Abdoulaye Diabaté, Rickard Ignell, Roch K. Dabiré, Olivier Gnankiné, Thierry Lefèvre

**Affiliations:** ^1^ Institut de Recherche en Sciences de la Santé (IRSS) Bobo‐Dioulasso Burkina Faso; ^2^ MIVEGEC, Université de Montpellier, IRD, CNRS Montpellier France; ^3^ Laboratoire d'Entomologie Fondamentale et Appliquée (LEFA), Unité de Formation et de Recherche—Sciences de la Vie et de la Terre (UFR‐SVT) Université Joseph KI‐ZERBO (UJKZ) Ouagadougou Burkina Faso; ^4^ Laboratoire Mixte International Sur les Vecteurs (LAMIVECT) Bobo‐Dioulasso Burkina Faso; ^5^ Institut Des Sciences et Techniques (INSTech—BOBO) Bobo‐Dioulasso Burkina Faso; ^6^ Unit of Chemical Ecology, Department of Plant Protection Biology, Disease Vector Group Swedish University of Agricultural Sciences Uppsala Sweden

**Keywords:** *Anopheles coluzzii*, *Anopheles gambiae*, behavioral choice, mosquito–plant interactions, nutritional ecology, survival

## Abstract

*Anopheles gambiae* and *Anopheles coluzzii* mosquitoes, two major malaria vectors in sub‐Saharan Africa, exhibit selectivity among plant species as potential food sources. However, it remains unclear if their preference aligns with optimal nutrient intake and survival. Following an extensive screening of the effects of 31 plant species on *An. coluzzii* in Burkina Faso, we selected three species for their contrasting effects on mosquito survival, namely *Ixora coccinea*, *Caesalpinia pulcherrima*, and *Combretum indicum*. We assessed the sugar content of these plants and their impact on mosquito fructose positivity, survival, and insemination rate, using *Anopheles coluzzii* and *Anopheles gambiae*, with glucose 5% and water as controls. Plants displayed varying sugar content and differentially affected the survival, sugar intake, and insemination rate of mosquitoes. All three plants were more attractive to mosquitoes than controls, with *An. gambiae* being more responsive than *An. coluzzii*. Notably, *C. indicum* was the most attractive but had the lowest sugar content and offered the lowest survival, insemination rate, and fructose positivity. Our findings unveil a performance–preference mismatch in *An. coluzzii* and *An. gambiae* regarding plant food sources. Several possible reasons for this negative correlation between performance and preference are discussed.

## Graphical abstract

This study investigated the plant preferences of females *Anopheles gambiae* and *Anopheles coluzzii*, two key malaria vectors in sub‐Saharan Africa. We found a mismatch between their preference for certain plants, such as *Combretum indicum*, and their actual performance in terms of survival, insemination rate, and sugar intake. These findings not only highlight the intricate relationships between mosquitoes and plants but also hold significance for understanding the ecology and evolution of foraging behaviors in these malaria vectors.

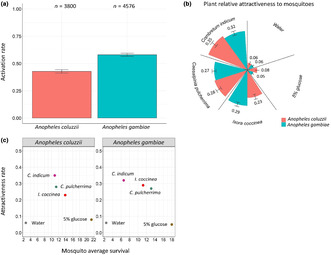

## 1 INTRODUCTION

According to optimal foraging theory, animals are expected to be able to discriminate and select among food resources that maximize nutrient intake and overall fitness, while considering the physiological and ecological trade‐offs associated with foraging (e.g., time and exposure to enemies), as well as ingestion and processing of food (Pyke et al., 1977; Stephens et al., 2007). In choice situations, numerous organisms—ranging from slime molds to humans—are capable of selecting food resources that provide the optimal balance of energy and nutrients, resulting in improved fitness (Berteaux et al., 1998; Dussutour et al., 2010; Griffioen‐Roose et al., 2012; Hill et al., 2019; Mayntz et al., 2005; Mittelbach, 1983; Schmidt et al., 2012; Simpson et al., 2004). As important pollinators, biocontrol agents, or pests, sugar‐feeding insects, such as bees, butterflies, parasitoid wasps, or adult flies, have received significant attention for their ability to self‐select food resources that enhance their survival and reproductive success (Fanson & Taylor, 2012; Hawley et al., 2016; Knauer & Schiestl, 2015; Lee et al., 2008; Mevi‐Schütz & Erhardt, 2005; Scheirs et al., 2004; Shahjahan, 1974; Van Rijn & Wäckers, 2016; Vaudo et al., 2016). Dipteran insects, including mosquitoes, black flies, sand flies, and biting midges, can transmit pathogens of major public and animal health concern. These vectors often visit flowers to gather nectar as a source of energy. However, it remains unclear whether the preference of insect vectors among different nectar sources correlates positively with the fitness benefits offered by the selected plants.

Mosquitoes are well known for feeding on blood, during which they can transmit pathogens responsible for devastating diseases, such as malaria, dengue fever, or Zika (World Health Organization, 2021). In most species, the females are hematophagous and blood is an essential source of protein to produce their eggs. A growing body of research, however, shows that, just like males, plant fluids, including floral and extra‐floral nectar, fruit, honeydew, and phloem or xylem sap, are an essential resource for mosquito females, with critical epidemiological implications (Stone & Foster, 2013). As the primary energy source for many important mosquito vectors, including *Culex*, *Aedes*, and *Anopheles*, plant fluids not only fuel flight and activity but can also be an important determinant of longevity and reproductive success (reviewed in Foster, 2022). Previous studies have shown that changes in the abundance or composition of particular flowering plant species can exert significant effects on the population dynamic and vectorial capacity of *Anopheles gambiae* sensu lato, the primary vector of the malaria parasite, *Plasmodium falciparum*, in Africa, through their impacts on the lifespan, susceptibility to pathogens, and reproductive output of individual mosquitoes (Ebrahimi et al., 2018; Gu et al., 2011; Hien et al., 2016).

Given the importance of floral resources for the biology of major malaria vectors, we still know surprisingly little about how intra‐ and interspecific variations in plant quality affect mosquito performance. Nectar, the main food resource that mosquitoes exploit from plants, is mostly composed of sugars (sucrose and its monomers, glucose and fructose), and, to a lesser extent, primary metabolites such as amino acids, lipids, vitamins, and proteins, and secondary metabolites, such as terpenes, alkaloids, and phenolics (Baker & Baker, 1983; Nicolson, 2022). Nectar quality varies among flowering plant species with respect to the concentration and composition of these sugars and other constituents (Barredo & DeGennaro, 2020; Palmer‐Young et al., 2019). Therefore, not all flowering plants are expected to offer suitable resources in terms of energy and nutrient intake and hence fitness benefits to malaria vectors, which, in turn, should likely lead to some degree of variability in their attractiveness to mosquitoes. Although several studies have examined flower suitability by measuring the fitness components of individual mosquitoes provided with a variety of plant species, only few have attempted to establish a connection with mosquito foraging preferences (Foster, 2022).

Mosquitoes rely on the integration of olfactory, visual, and gustatory cues to locate and select their host plants (Barredo & DeGennaro, 2020; Foster, 2022). Based on observations gathered from field, semi‐field, and laboratory settings, it appears that, although malaria vectors can use a wide variety of plant species as food sources, they display some degree of selectivity among the different plant species available in their natural habitats (Gouagna et al., 2010, 2014; Manda, Gouagna, Nyandat, et al., 2007; Müller et al., 2010; Nikbakhtzadeh et al., 2014; Nyasembe et al., 2012, 2018). To the best of our knowledge, only three studies have investigated whether *An. gambiae s.l*. exhibit a significant preference for the plant species that best support nutrient intake, survival, and/or fecundity (i.e., positive performance–preference relationships). First, among three flowering plant species in La Reunion Island, male *Anopheles arabiensis* were observed to accumulate greater levels of energy reserves, including sugar, lipids, and proteins, when feeding on their most preferred plant species (Gouagna et al., 2014). Second, following the classification of 13 plant species according to their attractiveness to female *An. gambiae* in Kenya (Manda, Gouagna, Nyandat, et al., 2007), mosquitoes were fed with one of each of five of the most attractive plants and one of the least attractive (Manda, Gouagna, Foster, et al., 2007). Although four of the five most attractive plants provided greater female survival and fecundity compared to the least attractive species, the highly attractive *Parthenium hysterophorus* provided low survival and fecundity (Manda, Gouagna, Foster, et al., 2007). Third, among four plant species that were highly attractive to *An. gambiae* females (Nikbakhtzadeh et al., 2014), three exhibited high sugar content and led to greater survival rates, while *P. hysterophorus* acted as a deceptive trap, causing high mortality probably associated with low sugar content (Nikbakhtzadeh et al., 2016). Collectively, these results suggest that, with the exception of *P. hysterophorus*, mosquitoes obtain greater fitness benefits when feeding on their preferred plant species, presumably due to the high sugar content present in these preferred plants. While the previous body of research was carried out in La Reunion and Kenya using *An. gambiae* and *An. arabiensis*, comparable studies in other parts of Africa and on other important vectors, such as *An. coluzzii*, are lacking. Expanding our understanding of the nutritional ecology of the major malaria vectors in Africa will provide insights into the factors that shape vectorial capacity in different endemic settings. This may also contribute to the identification of new attractive odor blends that could be used, for example, in the development of sugar baits/traps.

To gain further perspectives on the nature of malaria vector–plant interactions, we tested performance–preference relationships in a series of laboratory experiments in Burkina Faso using both *An. gambiae* and *An. coluzzii*, two primary vectors of *P. falciparum* in West Africa. We hypothesized that mosquito females would exhibit a significant selective behavior for the plant species that best support their survival and that feeding preference could be mediated by sugar intake and sugar content in plants. First, we screened the effects of 31 flowering plant species on *An. coluzzii* and selected three species with contrasting effects on mosquito survival. Second, we used no‐choice feeding assays to assess whether the consumption of these three plants affected the survival and sugar intake of *An. gambiae* and *An. coluzzii* females, using water and a 5% glucose solution as controls. Third, we quantified the sugar content of these three plant species. Owing to its effect on the body condition of both males and females, the variability in sugar quality among plant species can cause a proportion of females to remain uninseminated and therefore reduce the egg output of a population. Fourth, we therefore assessed the effect of the consumption of each of these three plant species by males alone, females alone, or both sexes on the insemination rate. Fifth, we performed multiple‐choice behavioral assays to determine whether females preferentially select plant species that offer higher fitness benefits in terms of sugar intake, insemination rates, and/or survival.

## 2 METHODS

### 2.1 Mosquito strains

Laboratory‐reared *An. coluzzii* and *An. gambiae* were obtained from outbred colonies established in 2019, which have since been repeatedly replenished with wild‐caught gravid females collected in the Vallée du Kou (11°23′N, 4°24′W) and Soumousso (11°04′N, 4°03′W), respectively, in southwestern Burkina Faso, and identified by SINE PCR (Santolamazza et al., 2008). Females were maintained on rabbit blood by direct feeding (protocol approved by the national committee of Burkina Faso; IRB registration #00004738 and FWA 00007038) for egg production. Larvae were reared in 1 L of tap water in plastic trays and fed daily with TetraMin® Baby Fish Food (Tetrawerke, Melle, Germany) until adulthood. The adult mosquitoes were held in 30 cm × 30 cm × 30 cm mesh‐covered cages under standard controlled conditions (27 ± 2°C, 70 ± 5% RH, and at a 12 h:12 h light:dark rhythm), and emerged males and females were fed daily with 5% glucose. This solution was prepared using D‐(+)‐glucose powder, Sigma‐Aldrich®, and distilled water.

### 2.2 Interspecific plant effects on mosquito performance

#### 2.2.1 Experiment 1.1: Screening of plant species that differentially support mosquito survival

The effect of 31 flowering plant species (Figure S1) on the survival of *An. coluzzii* mosquitoes was evaluated. The plants were selected based on their presence around human dwellings and public areas of the city of Bobo‐Dioulasso and the village of Farako‐ba, two localities in western Burkina Faso. The flowers were collected daily between 3 p.m. and 4 p.m. and offered to mosquitoes in 30 cm × 30 cm × 30 cm mesh‐covered cages at 5 pm. While wilted or dry flowers were discarded, flower age was not specifically controlled. This better reflects natural conditions, where mosquitoes encounter a wide range of flower ages. Between 7 and 10 freshly cut stems of flowering plants were arranged in a bouquet (with leaves removed) and introduced into the cages. The base of the bouquet was wrapped in moistened paper towels and covered with an aluminum sheet so that mosquitoes had no access to the moistened paper (Hien et al., 2016; Paré et al., 2022). A 5% glucose solution was used as a positive control by soaking a cotton pad with this solution and placing it on top of the control cage. The flower bouquets and the 5% glucose cotton pad were changed daily at 5 p.m. Disposable plastic cups (20 cL) containing about 30–40 pupae (males and females) were randomly assigned to one plant species. Adult mosquitoes (males and females with equal sex ratio, exact sample sizes are indicated in the legend of the corresponding figure below) emerging from these pupae were kept for 6 consecutive days on their assigned diet (one of four flowers or the glucose control solution). Mosquito survival was recorded from day 1 to day 6 post‐emergence. This consisted of counting dead mosquitoes daily, regardless of sex, between 4 and 5 p.m., and removing them from the cages. A specific permit for the sampling of the 31 plant species was obtained from the Ministère de l'Environnement, de l'Energie, de l'Eau et de l'Assainissement des Hauts‐Bassins of Burkina Faso. All plant species were identified by a botanist from the “Institut de Recherche pour le Développement” followed by an independent confirmation by a phytoecologist from the University Joseph KI‐ZERBO/University Center of Ziniaré Burkina Faso according to a catalog done by Thiombiano et al. (2012).

To further investigate the influence of plant species on mosquito survival rate, insemination rate, and sugar positivity, two experiments were conducted.

#### 2.2.2 Experiment 1.2: survival, cold‐anthrone tests, determination of the degree Brix, and the total sugar content

**FIGURE 1:**
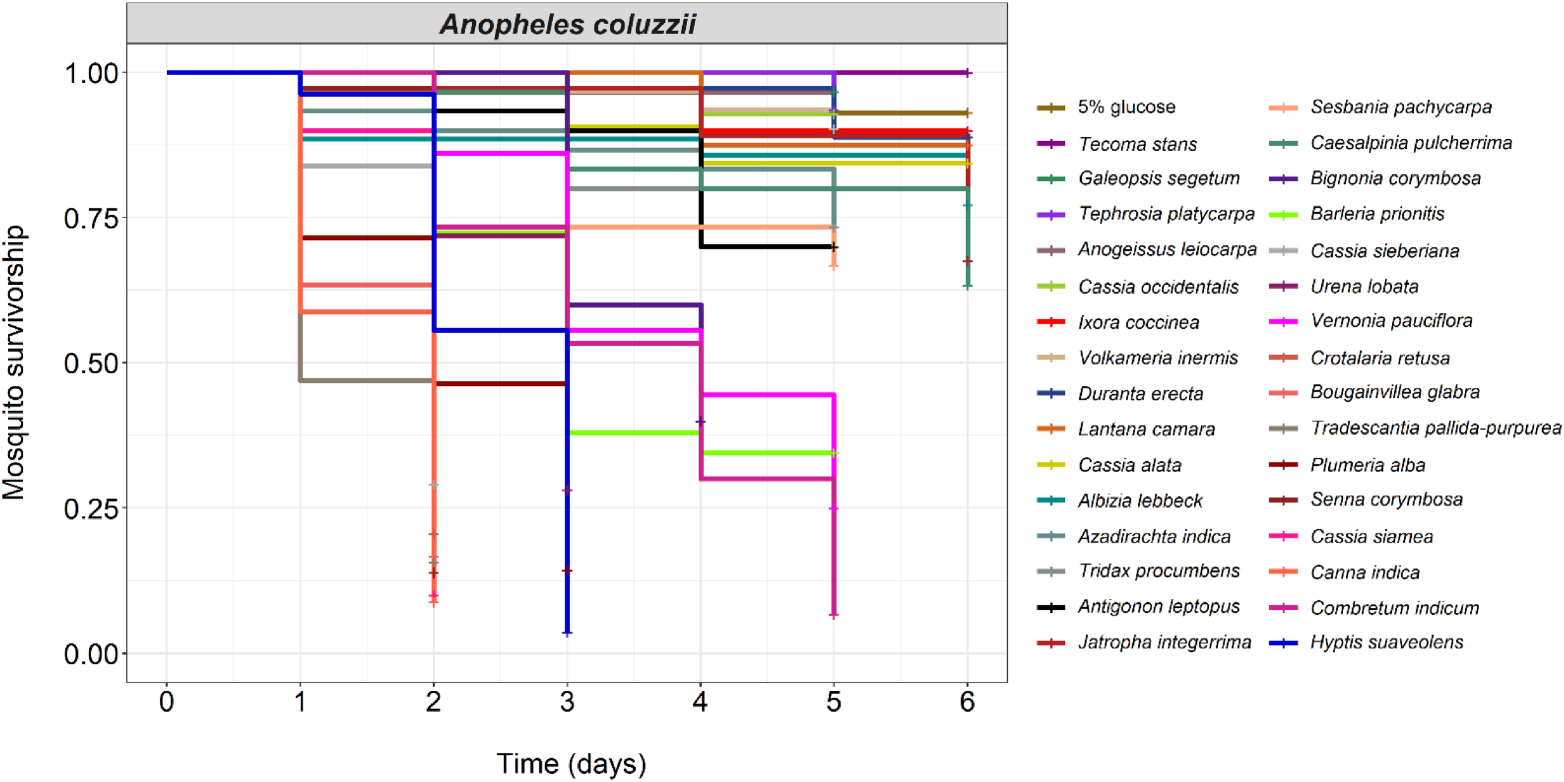
The effect of 31 plant species and a 5% control glucose solution on the survival of *Anopheles coluzzii*. Kaplan–Meier curves represent the proportion of live mosquitoes over time for each treatment. Between 27 and 37 emerging mosquitoes (males and females, mean ± SE: 31.25 ± 0.45, median: 30) were maintained on one of the treatments, and survival was recorded from day 1 to day 6 post‐emergence.

Based on the screening Experiment 1.1, three plant species were selected for their contrasting effects on mosquito survival (ranging from positive to negative effects on survival, Figure 1, Table S1), *Ixora coccinea* L. (Rubiaceae), *Caesalpinia pulcherrima* (L.) Sw. (Fabaceae‐Caesalpinioideae), and *Combretum indicum* (L.) Jongkind (Combretaceae*). Ixora coccinea* and *C. pulcherrima* provided high and medium mosquito survival, respectively, whereas *C. indicum* induced poor survival. The other species provided equivalent survival to *I. coccinea* but were not selected for the subsequent experiments because they were less abundant and available than *I. coccinea* at the time of the experiments. This was also true for the other species that provided equally poor survival as *C. indicum*. Specimens of *C. pulcherrima*, *C. indicum*, and *I. coccinea*, were deposited in the herbarium of the Nazi Boni University, Bobo‐Dioulasso, Burkina Faso, under the identification numbers UNB‐947, UNB‐948, and UNB‐949, respectively. All three species are exotic, widely cultivated ornamental plants in cities of Burkina Faso. *Ixora coccinea*, the jungle geranium, and *C. indicium*, the Rangoon creeper, are native to tropical Asia. *Caesalpinia pulcherrima*, the peacock flower, has a (sub‐) tropical distribution but its exact origin remains unclear (Datiles & Acevedo‐Rodríguez, 2014).

To further investigate the effect of these three plant species on the survival of malaria vectors, unlike the screening Experiment 1.1, both *An. coluzzii* and *An. gambiae* were used, and males and females were distinguished in this experiment. A total of 20 cages were used for this test (2 cages per treatment and species) each containing 130 pupae of both sexes. Upon emergence, males and females of each mosquito species were maintained together on one of five treatments: *I. coccinea*, *C. pulcherrima*, *C. indicum*, and water (negative control), and 5% glucose solution (positive control) (Figure S2). Mosquitoes were exposed to these treatments in the same manner and timing as in the screening Experiment 1.1. The mortality of males and females was monitored every day between 4 p.m. and 5 p.m. until all mosquitoes were dead.

In parallel with the longevity experiment, 10 additional 20 cm × 20 cm × 20 cm cages (1 cage per treatment and species) were set up to perform cold‐anthrone tests (Van Handel, 1972) (Figure S3). One‐hundred‐and‐ten pupae of *An. gambiae* or *An. coluzzii* were introduced into each cage at 9 a.m. The following day after emergence, at 5 p.m., the mosquitoes were provided with one of the five treatments (water, 5% glucose, *I. coccinea*, *C. pulcherrima*, and *C. indicum*) as described above. On the second day after emergence, at 8 a.m. (i.e., after 15 h of exposure to the treatments), all mosquitoes were aspirated to determine the proportion of fructose‐positive mosquitoes.

The °Bx and the total sugar content of the flowers of *I. coccinea*, *C. pulcherrima*, and *C. indicum* were determined in the biochemistry and microbiology laboratory of the Département Technologie Alimentaire of the Institut de Recherche en Sciences Appliquées et Technologies of Bobo‐Dioulasso. The °Bx is the mass fraction of sucrose in a liquid, representing the percentage by mass of soluble dry matter in the solution. One degree Brix is equivalent to 1 gram of sucrose per 100 g of solution. A hand‐held refractometer (Atago, ATC, Tokyo, Japan) with a scale of 0%–32%, and an accuracy of 0.2%, was used for the determination of °Bx (Harrill, 1998). Briefly, the flowers of each plant species were crushed using an electric grinder, type A11 basic (IKA®‐Werke GmbH & Co. KG, Staufen, Germany). One gram of crushed material of each species was wrapped in a net and squeezed to obtain a liquid solution. The refractometer was calibrated by placing two drops of distilled water on the main prism and was then cleaned and dried after each calibration. The solutions obtained by grinding were then placed on the refractometer prism. For each plant species, three measurements were made, each on different dates, and using different plant individuals.

Total sugar content was determined according to the sulfuric orcinol colorimetric method (Compaore et al., 2020). In the presence of concentrated sulfuric acid and at high temperatures, the hexoses and pentoses of the medium undergo a thorough internal dehydration, followed by a cyclization leading to the formation of furfural and 5‐hydroxymethylfurfural derivatives, reacting with orcinol to give a yellow–red complex. This allows the total sugar concentration of the sample to be monitored by reading the absorbance at 510 nm. At 6 am, flowers of each plant species were collected and brought back to the laboratory where they were immediately crushed by species using an electric grinder. One gram of crushed material from each species was taken and introduced into a 100 mL volumetric flask to which 5 mL of distilled water was added. The mixture was put under magnetic stirring for 10 min and the volume was completed to 100 mL with distilled water. One milliliter of each solution was introduced into test tubes (i.e., two tubes for each individual plant species) to which 2 mL of sulfuric orcinol reagent and 7 mL of 60% H_2_SO_4_ solution were added. The mixture was incubated in a boiling water bath for 20 min, placed under ambient temperature in the dark for 45 min, and then in natural light for 10 min. Then, a dilution was performed by adding 2 mL of 60% H_2_SO_4_ in 1 mL of each sampled solution. The optical densities of each diluted solution were read at 510 nm using a spectrophotometer (V‐1100D spectrophotometer, J.P. SELECTA, Barcelona, Spain). Sugar concentrations were determined using a standard curve, with D‐glucose as the reference sugar. The total sugar content of each species was expressed in D‐glucose equivalent as g/100 g fresh material (Compaore et al., 2020).

#### 2.2.3 Experiment 1.3: Survival and insemination

To evaluate the effect of plant treatment on mosquito survival and insemination rates, three assays, each with a different design (Figure S4), were conducted in parallel using *An. coluzzii* and *An. gambiae*. The three assays were replicated three times (i.e., a total of three experimental replicates were performed). The objective of design 1 was to determine whether the treatments, provided to both males and females, can influence sexual performance, as measured by female insemination rate. Design 2 aimed to assess the impact that the plants might have on the ability of females to get inseminated by males. In contrast, design 3 tested the effect of treatments on the ability of males to inseminate females.

- Design 1: Circa 30 newly emerged males and 30 newly emerged females were introduced into 20 cm × 20 cm × 20 cm cages and kept together for 5 days on one of four treatments: *I. coccinea*, *C. pulcherrima*, *C. indicum*, and 5% glucose solution (Figure S4a). Sample sizes varied slightly across species and replicates according to mosquito availability in the insectary (the exact sample sizes for each treatment, species, and replicates are indicated in Table S2).
- Design 2: Circa 30 newly emerged females were kept in 20 cm × 20 cm × 20 cm cages and fed daily with one of the four treatments. Three days later at 8 am, circa 30 males of the same age as the females, and previously fed on a 5% glucose solution for 3 days, were introduced into the female cages. Males and females were then kept together for 2 days on their assigned treatment (Figure S4b).
- Design 3: This design was similar to design 2 except that circa 30 newly emerged males, instead of females, were fed daily with one of the four treatments. On the morning of the 3rd day at 8 am, circa 30 females, previously fed on a 5% glucose solution for 3 days, were introduced into the male cages. Males and females were kept together for 2 days on their assigned treatment (Figure S4c).

Of particular note, males and females were housed together for 5 days in design 1, while in designs 2 and 3, the duration of contact between males and females was 2 days only. For each assay, females and males were at a ratio of 1:1 (Helinski et al., 2008; Stone et al., 2009) and plants were replaced every day with fresh materials in the same manner and timing as in the previous experiments. On the 5th day of the experiment at 8 a.m., the remaining females were retrieved and anesthetized at −20°C for 3 min. Spermathecae were dissected under a stereomicroscope (LEICA® S9E, Wetzlar, Germany) in a drop of distilled water and mounted under a coverslip. A gentle pressure was exerted on the coverslip with dissecting forceps to rupture the spermatheca, which was then observed under a compound light microscope (LEICA DM1000 LED, Germany) at 400× magnification to assess the insemination status (Figure S5). In all assays, mosquito mortality was recorded every day from 8 a.m. until day 5, and all remaining live male and female mosquitoes (used for spermatheca dissection) were considered in the survival analysis and given a censoring indicator of “0”. However, when either sex was first maintained on 5% glucose for 3 days (i.e., males in design 2 and females in design 3), then survival was monitored only from day 3 post‐emergence.

### 2.3 Mosquito behavioral response to plant species

Behavioral analysis was conducted in a multiple‐choice experimental device consisting of four large insect release cages (1 m × 1 m × 1 m) set‐up in a climate‐controlled room (29°C, 70 ± 5% RH). Each large release cage contained five smaller cages of 15 cm × 15 cm × 15 cm, each housing one of the five treatments (traps) (Figure S6a,b). The mesh screen of these traps was raised 3 cm from the ground to allow mosquitoes to enter (Figure S6c).

On the day of the behavioral test at 8 a.m., between 16 and 108 females of *An. coluzzii* (mean: 59.38 ± 1.20, median: 60.50) and between 35 and 109 females of *An. gambiae* (mean: 71.50 ± 0.91, median 70.00), aged between 1 and 4 days, and previously maintained on 5% glucose were aspirated and introduced into cardboard cups (four cups per species). The number of mosquitoes introduced into the cups (sample sizes) varied across behavioral tests (replicates) depending on mosquito availability in the insectary. To examine possible differences in behavioral responses between *An. gambiae* and *An. coluzzii*, two cups (one of each species) were released simultaneously into each large release cage. Females of both species were therefore exposed to the same flower bouquets, hence preventing the possible confounding effect of mosquito species and individual plant factors. For this purpose, one of the two mosquito species was marked with colored powder (Luminous Powder Kit, Bioquip Products Inc 2321 Gladwick Street Rancho Dominguez, CA 90220, USA). Marking was alternated among species, cages, and replicates to avoid confounding factors. The release of one cup of unmarked *An. gambiae* and one cup of marked *An. coluzzii* simultaneously in the same cage (and vice versa in other cages) allowed the species to be distinguished. Because mosquito number (varying sample sizes) and color marking might influence behavioral response, density and color were considered in the statistical analyses. A cotton pad soaked with water was placed on the mosquito cups, which were then kept under insectary conditions (27 ± 2°C and 70 ± 5% RH) prior to the test.

At 3.30 p.m. on the day of the behavioral test, flowers of *I. coccinea*, *C. pulcherrima*, and *C. indicum* were collected and made into a flower bouquet as described above, and then positioned in the small trap cages. In addition, two traps were baited with either 5% glucose cotton pads or water pads. These cotton pads were placed on a 20 cL disposable plastic cup positioned upside down on the bottom of the trap cages. The 5% glucose and water control treatments do not emit volatiles and serve the specific purpose of validating the device and ensuring the reliability of our results. A total of 20 small trap cages were used during each behavioral test (5 traps × 4 large cages). Each of these traps contained the flower bouquet of *I. coccinea*, *C. pulcherrima*, or *C. indicum*, or the 20 cL disposable cup holding either the 5% glucose cotton pad or the water cotton pad (Figure S7). The position of the traps was alternated randomly within the cages and among the 16 releasing nights (replicates).

At 6 p.m., *An. gambiae* and *An. coluzzii* contained in either of two cups (starved of sugar for a period of 10 h) were released simultaneously into one of the four release cages and were allowed 12 h to make a choice between treatments. Mosquitoes that were attracted to a treatment entered the cage trap through the 3 cm gap. With this configuration, the possibility that mosquitoes that entered a given trap managed to exit and remained in the release cage or visited another trap cannot be excluded. The following day the mosquitoes were collected. First, the odor trap nets were gently lowered and then tied to prevent the trapped mosquitoes from escaping. Mosquitoes that did not make a choice, that is, those remaining in the large release cages, were aspirated and stored in cardboard cups. Then, the nets of the large cages were removed and the caught mosquitoes were aspired from the small trap cages. These mosquitoes were placed in disposable plastic cups corresponding to their treatment and release cage identity. All aspirated mosquitoes were anesthetized at −20°C for counting by species (based on the color), by large release cage, and by trap cage (treatment). The 12 h duration of the behavioral trial was chosen on the basis of previous studies, which revealed that sugar feeding in both male and female *An. gambiae* followed a largely unimodal crepuscular/nocturnal diel rhythm (Gary & Foster, 2006).

The behavioral assay was repeated 16 times (16 nights) for a total of 64 choice episodes (16 nights × 4 large cages) for each mosquito species. Two traits were measured to analyze mosquito behavioral responses:

- activation rate, which is the number of mosquitoes caught in all traps out of the total number of mosquitoes released and
- plant relative attractiveness, which is the number of mosquitoes caught in each odor trap out of the total number of mosquitoes caught in all odor traps.

### 2.4 Statistical analysis

All statistical analyses were performed using R software (version 4.0.5) (R Core Team, 2023). Cox proportional hazard model (“coxph” function of the “survival” library version 3.2‐10; Therneau, 2021) with censoring was performed to test the effect of diet (32 levels) on mosquito survivorship (Experiment 1.1). A Cox's proportional hazard mixed regression model (“coxme” library version 2.2‐16; Therneau, 2020) without censoring and with cage (20 levels) set as a random effect was performed to test the effect of treatment (five levels: water, 5% glucose, *I. coccinea*, *C. pulcherrima*, and *C. indicum*), mosquito species (two levels: *An. coluzzii* and *An. gambiae*), sex (2 levels), and their interactions on mosquito survivorship (Experiment 1.2). Logistic regression by generalized linear model (GLM, quasibinomial errors, logit link) was used to test the effect of treatment, mosquito species, sex, and their interactions on the proportion of mosquitoes positive to cold anthrone (Experiment 1.2). The effect of treatment (four levels: 5% glucose, *I. coccinea*, *C. pulcherrima*, and *C. indicum*), mosquito species (two levels), design (three levels), sex (two levels), and their interactions on mosquito survivorship was analyzed using a censored Cox's proportional hazard mixed regression model (“coxme” library version 2.2‐16; Therneau, 2020) with replicate (three levels) set as a random effect (Experiment 1.3). The effect of treatment, mosquito species, design, and their interactions on insemination rate was analyzed using a logistic regression by generalized mixed linear models (GLMM, binomial errors, logit link; “lme4” library version 1.1‐32; Bates et al., 2015) with replicate (three levels) set as a random effect (Experiment 1.3). A binomial GLMM was also used to test the effect of species (two levels), coloration (two levels: uncolored and colored), density (the number of mosquitoes released in the large cages, log‐transformed), and relevant two‐ways interactions on mosquito activation (Experiment 2). A mixed‐effects multinomial logistic regression model (“mblogit” function of the “mclogit” library version 0.9.6; Elff, 2022) was used to explore the effect of species (two levels), coloration (two levels), density (log‐transformed), and relevant two‐ways interactions on plant relative attractiveness to mosquitoes (Experiment 2). The relative odds ratios were then derived to compare the likelihood of mosquitoes choosing one treatment over another. In these two mixed models (binomial GLMM for mosquito activation and multinomial GLMM for plant relative attractiveness), the cage (four levels) was nested within night (16 levels) and considered together as nested random effects.

The “Anova” function from the “car” library version 3.1‐1 (Fox & Weisberg, 2019) was used to estimate the significance of terms. This function computes type II or III analysis‐of‐variance tables, including Wald chi‐square tests for assessing the significance of the fixed effects in our GLMMs. For the attractiveness analysis (multinomial GLMM using mclogit), the best model was selected based on the Akaike information criteria (AIC). Multiple pairwise post hoc tests were performed to compare each level of the treatment when the latter was significant using the “emmeans” function (with Tukey HSD adjustment) of the “emmeans” library version 1.5.5‐1 (Lenth, 2021).

## 3 RESULTS

### 3.1 Interspecific plant effects on mosquito performance

#### 3.1.1 Experiment 1.1: Screening of plant species that differentially support mosquito survival

Mosquito survival rate varied widely among plant species (LRT $X_{31}^{2}$ = 869.14; *p* < .001, Figure 1, Table S1). On day 6 post‐emergence, when mortality monitoring was stopped, the survival rate of mosquitoes fed with the 5% control glucose solution was 93 ± 0.1%. On the basis of multiple pairwise post hoc comparisons, the survival rate of mosquitoes fed with either *Tecoma stans* (100 ± 0.00%), *Galeopis segetum* (97 ± 0.07%), *Tephrosia platycarpa* (94 ± 0.09%), *Cassia occidentalis* (93 ± 0.1%), *Anogeissus leiocarpa* (93 ± 0.1%), *Volkameria inermis* (90 ± 0.11%), *I. coccinea* (90 ± 0.11%), *Duranta erecta* (89 ± 0.11%), *Lantana camara* (88 ± 0.12%), *Cassia alata* (84 ± 0.14%), *Albizia lebbeck* (77 ± 0.16%), *Azadirachta indica* (73 ± 0.18%), *Tridax procumbens* (73 ± 0.18%), *Antigonon leptopus* (70 ± 0.20%), *Jatropha integerrima* (68 ± 0.18%), *Sesbania pachycarpa* (67 ± 0.21%), or *C. pulcherrima* (63 ± 0.22%) was similar to that of mosquitoes fed with the 5% glucose solution (Table S3). In contrast, plant species, including *Bignonia corymbosa* (40 ± 28%), *Barleria prionitis* (34 ± 29%), *Cassia sieberiana* (29 ± 30%), *Urena lobata* (28 ± 29%), *Vernonia pauciflora* (25 ± 28%), *Crotalaria retusa* (21 ± 30%), *Bougainvillea glabra* (17 ± 33%), *Tradescantia pallida‐purpurea* (16 ± 32%), *Plumeria alba* (14 ± 34%), *Senna corymbosa* (14 ± 30%), *Cassia siamea* (10 ± 34%), *Canna indica* (9 ± 32%), *C. indicum* (7 ± 35%), and *Hyptis suaveolens* (4 ± 37%) negatively impacted mosquito survival compared to the 5% glucose solution (Figure 1, Table S3).

To assess performance–preference relationships, we subsequently selected three species that were abundant at the time of the following experiments and had contrasting effects on mosquito survival, namely *I. coccinea*, *C. pulcherrima*, and *C. indicum* (ranked according to their effect on survival from positive to negative).

#### 3.1.2 Experiment 1.2: Survival, cold‐anthrone tests, determination of the degree Brix, and total sugar content

##### Survival

**FIGURE 2:**
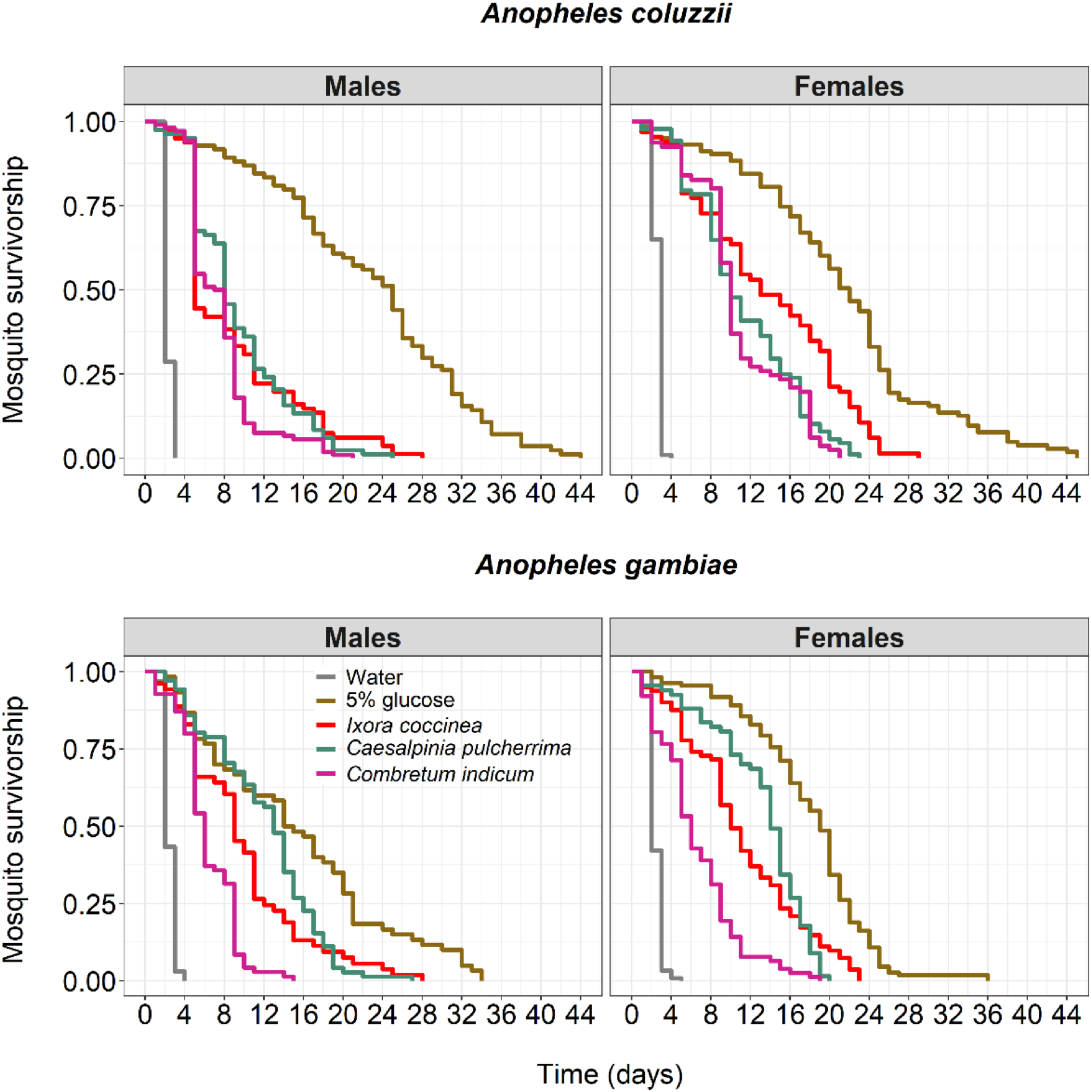
Effect of plant treatments on the survival of *Anopheles coluzzii* and *Anopheles gambiae* according to sex. Kaplan–Meier curves represent the proportion of live mosquitoes over time for each diet (water = negative control, 5% glucose solution = positive control). There were 130 pupae in each cage. The survival of males and females *An. coluzzii* and *An. gambiae* was monitored until all mosquitoes were dead.

While there was no species effect on mosquito survival (LRT $X_{1}^{2}$ = 2.52, *p* = .11, Figure 2, Tables S4 and S5), females exhibited a median survival time of 10 days, 4 days greater than that of males (LRT $X_{1}^{2}$ = 10.60, *p* = .001, Figure 2). Mosquito survival strongly varied among treatments (LRT $X_{4}^{2}$ = 861, *p* < .001, Figure 2, Tables S4 and S5), and all pairwise differences were significant except that between *I. coccinea* and *C. pulcherrima* (Table S6). In the absence of a food source, that is, water only, mosquitoes died within 3–5 days and were 135.65 times more likely to survive when kept in a 5% glucose solution rather than in water (Figure 2, Table 1). Compared to the water control, the chances of survival were 20.69, 38.19, and 42.60 times greater when mosquitoes were maintained on *C. indicum*, *C. pulcherrima*, and *I. coccinea*, respectively (Table 1). There was an interaction between treatment and species (LRT $X_{4}^{2}$ = 35.06, *p* < .001, Figure 2), with a survival difference between *C. indicum* and *C. pulcherrima* within *An. gambiae* but not within *An. coluzzii*. There also was a treatment‐by‐sex (LRT $X_{4}^{2}$ = 21.04, *p* < .001) and a species‐by‐sex (LRT $X_{1}^{2}$ = 4.04, *p* = .04) interaction. There was no three‐way interaction (Figure 2, Table S4).

**TABLE 1:** Risk of mosquito survival (hazard ratio) without censoring for each treatment relative to the control (water).

Treatment	Hazard ratio (lower 0.95 to upper 0.95)	*Z*	*p*‐Value
5% Glucose	135.65 (0.01–0.01)	35.41	**<.001**
*Ixora coccinea*	42.60 (0.02–0.03)	28.35	**<.001**
*Caesalpinia pulcherrima*	38.19 (0.02–0.03)	28.01	**<.001**
*Combretum indicum*	20.69 (0.04–0.06)	24.31	**<.001**

*Note*: Lower 0.95 to upper 0.95 represent the 95% confidence interval around the hazard ratio.

Bold values show *p*‐value  < .05.

##### Cold‐anthrone test

**FIGURE 3:**
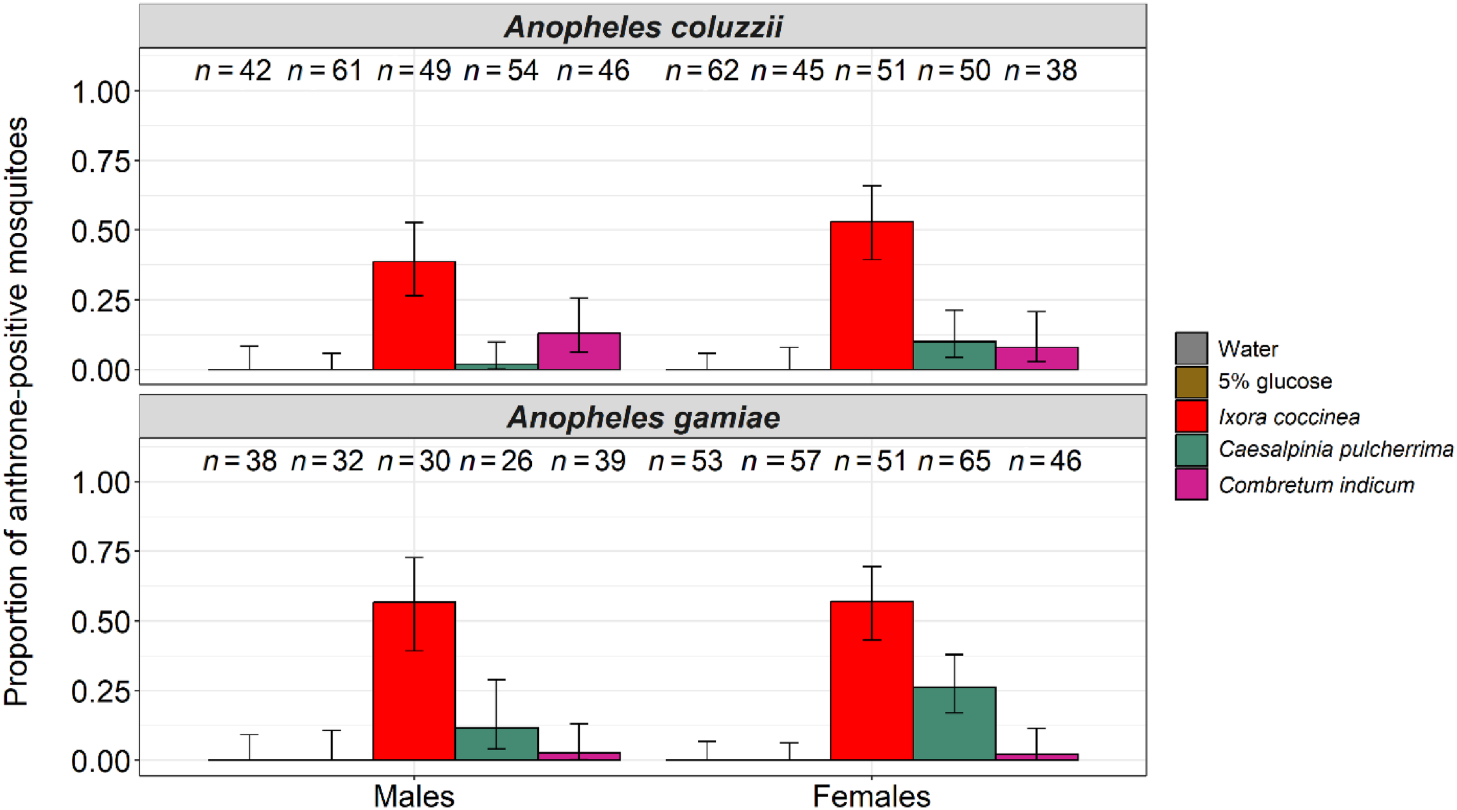
Proportion of *Anopheles coluzzii* and *Anopheles gambiae* (males and females) tested positive for fructose after exposure to one of five treatments: water, 5% glucose solution, *Ixora coccinea*, *Caesalpinia pulcherrima*, and *Combretum indicum* for 15 h for *An. coluzzii* and *An. gambiae*. The numbers above the barplots represent the sample size for each treatment. The error bars represent the variability of data with 95% confidence interval (±95% CI).

The anthrone test was carried out on all mosquitoes that emerged after 15 h of contact with the treatments. Overall, the proportion of fructose‐positive mosquitoes varied among treatments (LRT $X_{4}^{2}$ = 267.24, *p* < .001, Figure 3, Table S7), ranging from 0% in water and in glucose to 6 ± 0.15% in *C. indicum*, 13 ± 0.13% in *C. pulcherrima*, and 50 ± 0.10% in *I. coccinea*. Fructose positivity did not vary between species and between sexes (12 ± 0.08% and 16 ± 0.09% for *An. coluzzii* and *An. gambiae*, respectively, LRT $X_{1}^{2}$ = 2.78, *p* = .1; 11 ± 0.09%, and 16 ± 0.08% for males and females, respectively, LRT $X_{1}^{2}$ = 2.96, *p* = .09, Figure 3, Table S7). There was a significant interaction between treatment and species (LRT $X_{4}^{2}$ = 12.66, *p* = .01, Figure 3), with a higher proportion of fructose‐positive individuals in the *C. pulcherrima* treatment compared to *C. indicum* for *An. gambiae*, while no such difference was noted for *An. coluzzii*.

##### Determination of the degree Brix and the total sugars

Although there were only three observations per plant species, thus precluding statistical analysis, our results suggest that the °Bx of *C. pulcherrima* was higher than that of *C. indicum* and *I. coccinea* (Table 2). With respect to sugar content, only two observations per plant were available, but *C. indicum* demonstrated values half of that of *C. pulcherrima* and *I. coccinea* (Table 2).

**TABLE 2:** Mean °Bx and sugar content.

Plants	Mean °bx (±SE)	Mean sugar content (±SE)
*Ixora coccinea*	12.67 ± 0.77	8.06 ± 0.68
*Caesalpinia pulcherrima*	16.70 ± 1.65	8.35 ± 0.03
*Combretum indicum*	12 ± 1	4.19 ± 0.52

*Note*: The mean °Bx value was calculated based on three measurements taken from three different flower/plant individuals at different time intervals, whereas the mean sugar content was determined by averaging two measurements taken from the same plant during the same time period. Sugar content was expressed in g/100 g of fresh matter.

Abbreviation: SE, standard error.

#### 3.1.3 Experiment 1.3: Survival and insemination

##### Survival

**FIGURE 4:**
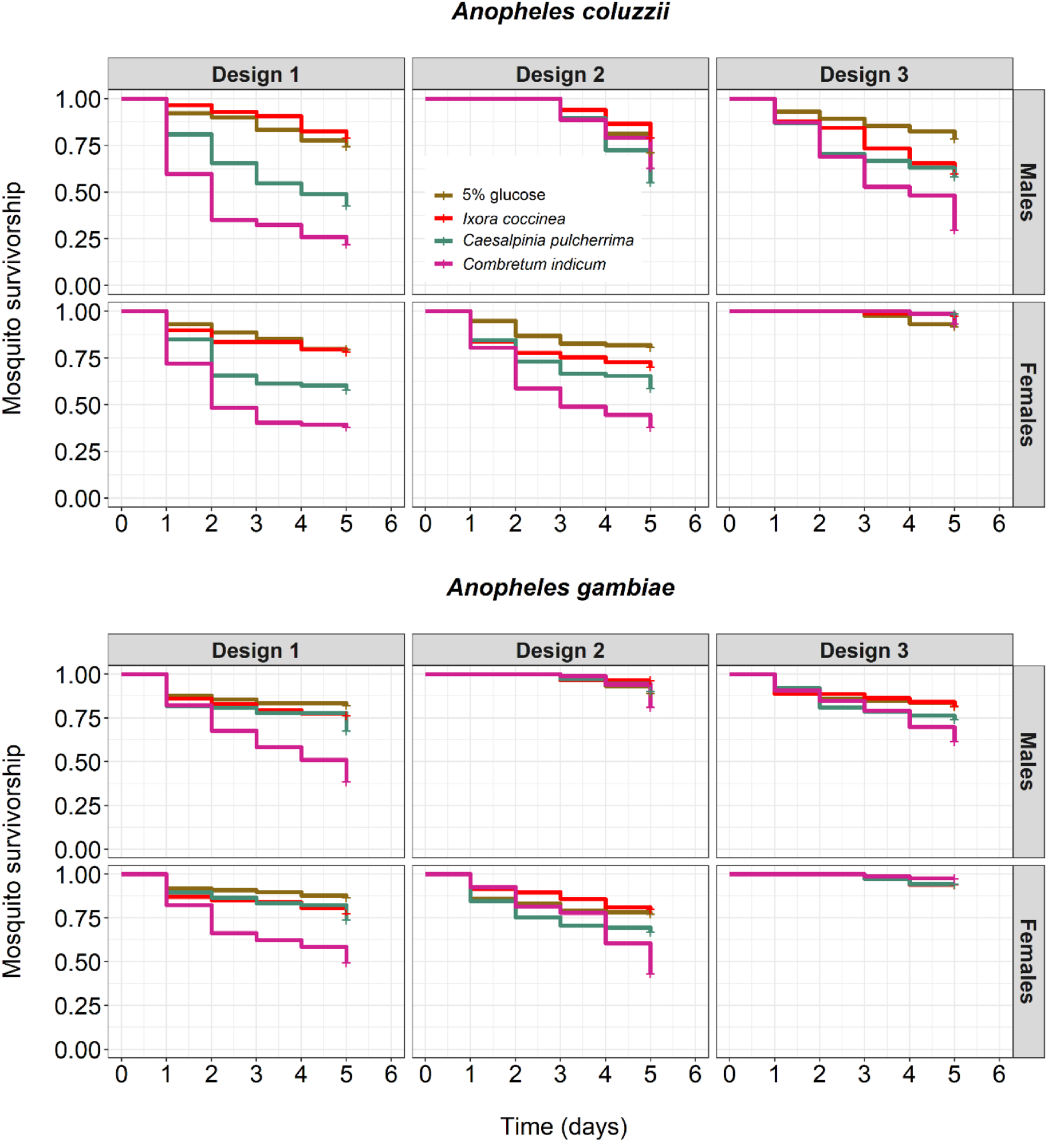
Effect of treatment, species, sex, and design on mosquito survival over three replicates. Design 1: males and females were kept together for 5 days on one of four treatments. Design 2: males were first fed 5% glucose solution for 3 days before being introduced with the females maintained on the treatments. Design 3: females were first fed 5% glucose solution for 3 days before being introduced with males maintained on the treatments. Kaplan–Meier curves represent the proportion of live mosquitoes for each treatment from day 1 to day 5 post‐emergence.

Mosquito survival on day 5 varied among treatments (LRT $X_{3}^{2}$ = 98.57, *p* < .001, Figure 4, Table S8), ranging from 82 ± 0.03% on 5% glucose to 81 ± 0.03% on *I. coccinea*, 69 ± 0.03% on *C. pulcherrima*, and 54 ± 0.04% on *C. indicum*. All pairwise differences were significant except that between glucose 5% and *I. coccinea* (Table S9). There were no survival differences between species (*An. coluzzii*: 67 ± 0.03%, *An. gambiae*: 77 ± 0.02%, LRT $X_{1}^{2}$ = 0.97, *p* = .32, Figure 4, Table S8), sex (male: 68 ± 0.02%, female: 75 ± 0.02%, LRT $X_{1}^{2}$ = 0.49, *p* = .48, Figure 4, Table S8), and design (design 1: 64 ± 0.03%, design 2: 72 ± 0.03%, design 3: 80 ± 0.02%, LRT $X_{2}^{2}$ = 0.99, *p* = .61, Figure 4, Table S8). The survival of males or females was improved when they were first maintained for 3 days on a 5% glucose solution (males design 2 and females design 3 in Figure 4). In addition, such maintenance on 5% glucose alleviated the differences caused by the treatments, resulting in a significant design by treatment interaction (LRT $X_{6}^{2}$ = 32.72, *p* < .001, Figure 4, Table S8). There was also a significant interaction among treatment, sex, and design (LRT $X_{6}^{2}$ = 24.59, *p* < .001, Figure 4, Table S8). All other interactions were non‐significant (Table S8). The results from this experiment confirm the two previous survival assays: mosquito survival on *C. indicum* was the worst regardless of sex and species. Separate analyses and figures were also produced for each of the three designs (Table S8 and Figure S8).

##### Insemination rate

**FIGURE 5:**
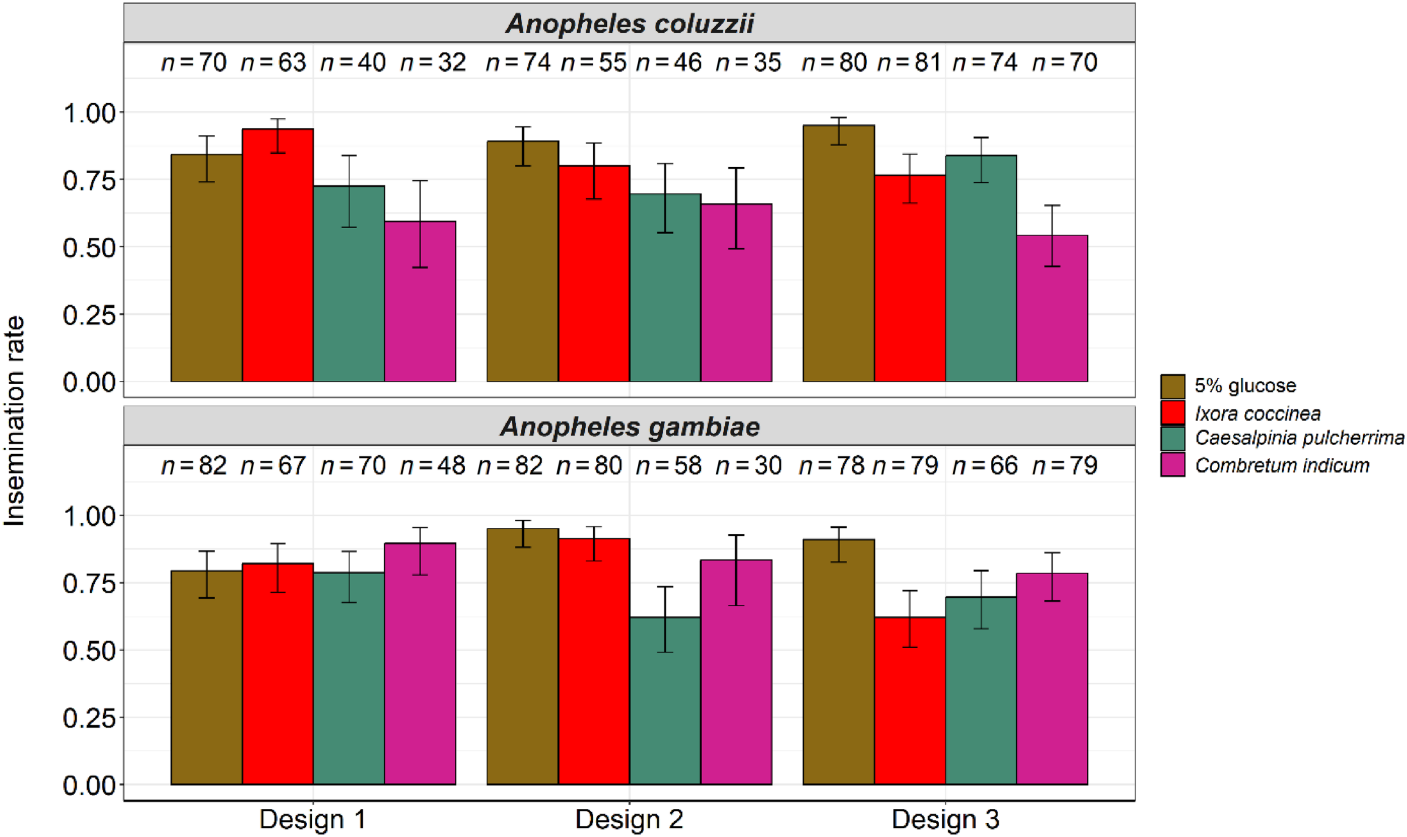
Effect of treatment, species, and design on insemination rate over three replicates. Treatments were 5% glucose control solution, *Ixora coccinea*, *Caesalpinia pulcherrima*, and *Combretum indicum*. The numbers above the barplots represent the sample size for each treatment. The error bars represent the variability of data with 95% confidence interval.

Female insemination rate varied among treatments (LRT $X_{3}^{2}$ = 25.38, *p* < .001, Figure 5, Table S10), with *C. indicum* causing the lowest insemination rate (71 ± 0.06%), followed by *C. pulcherrima* (73 ± 0.05%), *I. coccinea* (80 ± 0.04%), and 5% glucose (89 ± 0.03%) (Figure 5, Table S11). All pairwise differences were significant except that between *C. pulcherrima* and *C. indicum* (Table S12). There were no differences in insemination rates between species (*An. coluzzii*: 79 ± 0.03%, *An. gambiae*: 80 ± 0.03%, LRT $X_{1}^{2}$ = 1.16, *p* = .28, Figure 5, Tables S10 and S11). There was no effect of design, that is, feeding the treatments to males exclusively for 3 days (design 3), females solely for 3 days (design 2), or both sexes simultaneously for 3 days (design 1) had no effect on mosquito insemination rates (design 1: 81 ± 0.04%, design 2: 82 ± 0.04%, design 3: 77 ± 0.04%, and LRT $X_{2}^{2}$ = 3.97, *p* = .14, Figure 5, Tables S10 and S11). There were significant treatment‐by‐species (LRT $X_{3}^{2}$ = 15.60, *p* = .001, Figure 5, Table S10) and treatment‐by‐design (LRT $X_{6}^{2}$ = 16.06, *p* = .01, Figure 5, Table S10) interactions as well as a three‐way interaction among treatment, species, and design (LRT $X_{6}^{2}$ = 14.64, *p* = .02, Figure 5, Table S10). Separate analyses and figures were also produced for each of the three designs separately (Table S10 and Figure S9).

### 3.2 Mosquito behavioral response to plant species

A total of 3800 *An. coluzzii* and 4576 *An. gambiae* were released on 64 occasions over 16 nights (4 release cages/night), and activation rate and plant relative attractiveness were measured.

#### 3.2.1 Activation

**FIGURE 6:**
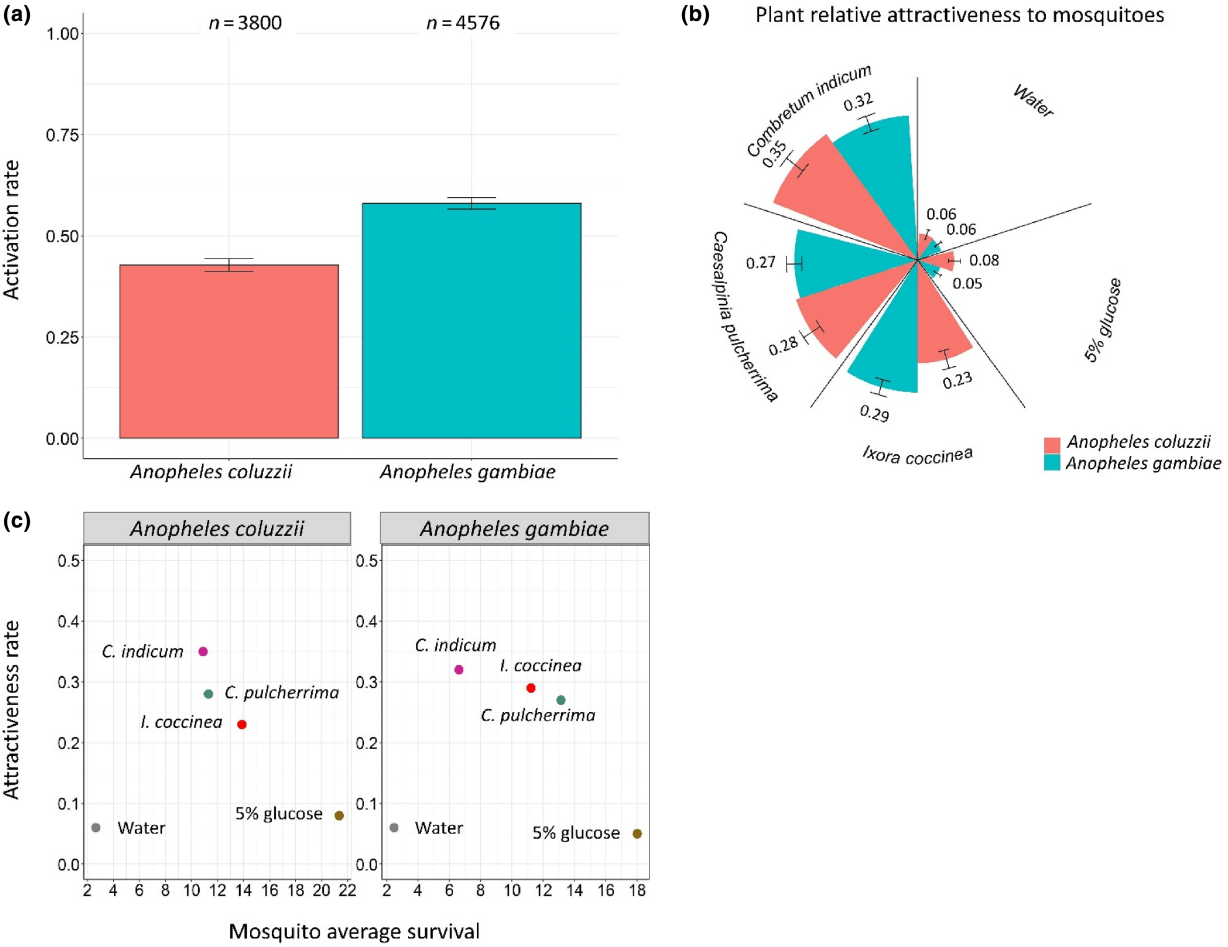
Mosquito behavioral response. (a) Activation rate of mosquitoes (i.e., number of mosquitoes caught in all traps out of the total number of mosquitoes released). An average of 59.38 (±1.20) *Anopheles coluzzii* and 71.5 (±0.91) *Anopheles gambiae* females were released in one of four cages from 6 p.m. to 6 a.m. The numbers above the barplots represent the total number of released mosquito. (b) Plant relative attractiveness to mosquitoes (i.e., number of mosquitoes caught in one trap out of the total number of mosquitoes caught in all traps). The numbers above the bars represent this attractiveness index. The error bars represent 95% confidence interval (±95% CI). The experiment was repeated over 16 nights leading to a total of 64 releases (4 cages × 16 nights). (c) Attractiveness index in relation to mosquito survival using mosquito average survival in Experiment 1.2.1. The dots represent the treatments.

Overall, the activation rate of *An. gambiae* (58% [0.57–0.59], with 2653 of the 4576 released *An. gambiae* flying into one of the five odor traps) was higher than that of *An. coluzzii* (43% [0.41–0.44], 1625 of 3800 released *An. coluzzii*) (LRT $X_{1}^{2}$ = 70.65, *p* < .001, Figure 6a). Color marking increased mosquito activation (LRT $X_{1}^{2}$ = 80.68, *p* < .001, Figure S10), regardless of mosquito species (i.e., no species‐by‐color interaction, LRT $X_{1}^{2}$ = 0.08, *p* = .78, Figure S10). There was no effect of density on mosquito activation (LRT $X_{1}^{2}$ = 2.68, *p* = .10). Figure S11 shows mosquito activation rates for each of the 16 nights.

#### 3.2.2 Attractiveness

Based on AIC, the minimum adequate model included species only as the main effect, that is, there was no influence of color marking, density, or interactions on plant relative attractiveness (Table S17). In particular, *An. coluzzii* females exhibited a greater preference for the 5% glucose solution and a lesser preference for *I. coccinea* compared to their *An. gambiae* counterparts (Figure 6b). Given the significant effect of mosquito species, separate analyses were conducted for each species to investigate their respective preferences among the five treatments. Table 3 shows the relative odds ratios between treatments for each mosquito species. First, the water and glucose treatments were equally attractive, but much less than the three plant species. Second, *C. indicum* was the most attractive plant to mosquitoes, with odds ratios of 6.6 and 6.8 for *An. coluzzii* and *An. gambiae*, respectively, compared to the control water treatment. Third, while the attractiveness of *C indicum* to mosquitoes was not significantly higher than that of *C. pulcherrima*, *An. coluzzii* showed a significant preference for *C. indicum* compared to *I. coccinea*. Figure S12 shows the relative attractiveness of treatments for each of the 16 nights.

**TABLE 3:** Relative odds ratios of attractiveness between treatments for each mosquito species.

	*Anopheles coluzzii*		*Anopheles gambiae*	
	Relative OR (95% CI)	*p*‐Value	Relative OR (95% CI)	*p*‐Value
Water/5% glucose	1.17 (0.65–2.11)	.61	0.85 (0.52–1.38)	.50
Water/*Caesalpinia pulcherrima*	5.22 (3.01–9.08)	**<.001**	5.17 (2.76–9.68)	**<.001**
Water/*Combretum indicum*	6.64 (3.86–11.41)	**<.001**	6.80 (3.67–12.60)	**<.001**
Water/*Ixora coccinea*	4.29 (2.55–7.23)	**<.001**	5.51 (3.21–9.46)	**<.001**
5% Glucose/*C. pulcherrima*	4 (2.46–6.48)	**<.001**	5.57 (3.00–10.35)	**<.001**
5% Glucose/*C. indicum*	5.09 (3.08–8.41)	**<.001**	7.27 (3.78–13.99)	**<.001**
5% Glucose/*I. coccinea*	3.27 (2.07–5.16)	**<.001**	5.91 (3.35–10.43)	**<.001**
*C. pulcherrima/C. indicum*	1.24 (0.83–1.85)	.30	1.27 (0.84–1.92)	.25
*C. pulcherrima/I. coccinea*	0.79 (0.53–1.18)	.25	1.04 (0.65–1.65)	.89
*C. indicum*/*I. coccinea*	0.64 (0.42–0.97)	**.03**	0.80 (0.51–1.24)	.31

*Note*: The relative odds ratios and 95% confidence interval were derived from the mixed‐effects multinomial logistic regression model by exponentiating the regression coefficients.

Bold values show *p*‐value  < .05.

Abbreviation: OR, odds ratio.

Finally, we explored the association between mosquito preference and performance (using average mosquito survival as a proxy) by plotting the index of relative attractiveness against mosquito survival (Figure 6c). Although the limited number of tested plant species precluded proper linear fitting, the relationship between plant attractiveness and mosquito survivorship tended to be negative, suggesting that *An. gambiae* and *An. coluzzii* did not prefer plant species that provided the best survivorship. This negative relationship between preference and performance was also obtained with our additional survival assay (Figures S13 and S14).

## 4 DISCUSSION

Findings presented in this study do not support the hypothesis that females of the major malaria vectors *An. gambiae* and *An. coluzzii* exhibit significant preferences for the plant species that best supports their fitness, and that this could be mediated by both mosquito sugar intake and sugar content in plants. Rather, among the three species tested, *C. indicum*, the most attractive plant species in the multiple‐choice assays, had the lowest sugar content and provided the lowest mosquito survival, insemination rate, and fructose positivity. Our results are inconsistent with the few previous studies suggesting that *An. gambiae* males and females are generally attracted to plants that provide abundant sugars, which in turn prolongs their survival (Gouagna et al., 2014; Manda, Gouagna, Foster, et al., 2007; Nikbakhtzadeh et al., 2016). Instead, we observed a mismatch between mosquito performance and preference, and in this respect, our results align with the exception found in the studies of Manda, Gouagna, Foster, et al. (2007) and Nikbakhtzadeh et al. (2016), wherein *P. hysterophorus* was found to be attractive despite providing little sugar and not extending mosquito survival.

A first possible explanation for this mismatch is that *C. indicum* (and *P. hysterophorus*) are deceptive flowers. Studies have demonstrated that mosquitoes are drawn to floral semiochemicals, such as linalool oxides (Nyasembe, Tchouassi, et al., 2015; Nyasembe & Torto, 2014). The main components detected in the extracts of flowers of *C. indicum* are, in fact, linalool oxides (Rout et al., 2008), and it is plausible that *C. indicum* would release the right amount of such semiochemicals, making it highly attractive to mosquitoes. Furthermore, while the relative importance of visual versus chemical cues in our multiple‐choice trap device is unknown, it is recognized that mosquitoes can sense colors, especially in the ultraviolet light ranges (Barredo & DeGennaro, 2020; Peach et al., 2019) In the context of pollinator–plant interactions, the chemical and visual floral stimuli are typically used to signal the presence of a food source, communicating the suitability of a plant for insects, that is, act as so‐called “honest signals” (Knauer & Schiestl, 2015). However, in the case of deceptive plants, these stimuli are a false promise of a reward that the plants do not actually provide (Heiduk et al., 2015; Jersáková et al., 2006). Through intricate olfactory, visual, and tactile cues, pollinators are tricked by these rewardless flowers, which ultimately leave them empty handed (Heiduk et al., 2015; Jersáková et al., 2006). In our specific case with *C. indicum*, we posit that the plant may primarily attract its intended pollinators while incidentally drawing non‐pollinating insects, including mosquitoes. Previous research has shown that *C. indicum* flowers undergo primary pollination by hawk moths during the white floral stage and by butterflies during the pink and red floral stages (Eisikowitch & Rotem, 1987; Yan et al., 2016). This observation aligns with the concept of pollination syndromes, wherein Sphingidae and Lepidoptera exhibit elongated proboscises adapted to the long receptacles of *C. indicum* flowers. Importantly, the proboscis of mosquito females is too short for effective pollination; nevertheless, they may engage in nectar theft by piercing the base of the receptacle and/or feeding on plant sap.

A related scenario proposed by Nikbakhtzadeh et al. (2016), in an effort to explain the paradox surrounding *P. hysterophorus*, would be that mosquitoes use the visual or olfactory cues to locate plant hosts, with no regard for the sugar content of the plants. This may be a reasonable assumption, as *C. indicum* (like *P. hysterophorus*) is an exotic species in Africa, allowing little time for *An. gambiae* to have evolved adaptive preferences for these introduced plants over local plants (Foster, 2022; Nikbakhtzadeh et al., 2016). In this study, the stimuli, including semiochemicals, involved in the attractiveness of *C. indicum* to mosquitoes was not determined. Further research is thus needed to characterize and isolate the mechanisms and stimuli contributing to floral preference. Besides fundamental interest, these mechanisms could be exploited to develop odor lures to trap or kill mosquitoes.

Second, the influence of sugar content alone may not fully account for the observed variations in survival among treatments. In particular, previous investigations on female specimens of *Aedes aegypti* and *Culex quinquefasciatus* suggest that amino acids present in nectar could enhance survival rates (Nyasembe et al., 2021; Vrzal et al., 2010). In contrast, the presence of toxic secondary compounds in nectars (Stevenson et al., 2017) might reduce mosquito survival (Hien et al., 2016, 2021; Nyasembe, Cheseto, et al., 2015). The chemical composition of the plant species utilized in this study has not been thoroughly characterized, and the presence or quantity of amino acids or secondary toxic compounds in nectars remains unknown. However, studies have revealed the presence of secondary compounds, such as terpenes, alkaloids, phenols, glycosides, and flavonoids, in flower or leaf extracts of these plant species (Anila & Hashim, 2019; Lim, 2014; Owolabi et al., 2022; Prasad et al., 2011; Rout et al., 2008). It is uncertain whether, in our no‐choice assays, the mosquitoes indeed consumed nectar rather than phloem or xylem fluids from tissue piercing, as previously observed (Foster, 2022; Manda, Gouagna, Nyandat, et al., 2007; Müller & Schlein, 2005). The possibility of mosquito tissue feeding and the existence of potentially toxic compounds in the plant fluids could partially explained why the 5% glucose solution, with an equivalent °Bx of 4.3, provided better mosquito survival compared to plants with flowers exhibiting higher °Bx (> 10). Nonetheless, the most plausible explanation for the observed favorable survival on the 5% glucose solution remains that despite its lower sugar concentration, it was readily accessible (via soaked cotton), highly fluid, and therefore easily ingested in unrestricted quantities.

Third, it is important to note that a resource is not limited to food alone; it can also include resting sites, shelter, and mating partners, among other things. For instance, the enemy‐free space hypothesis suggests that phytophagous insects may use certain host plants for refuge and defense against natural predators (Jeffries & Lawton, 1984; Singer et al., 2004). Accordingly, our findings suggest that the plant preference of *An. gambiae* and *An. coluzzii* may be influenced by factors beyond nutritional quality, such as the availability of favorable resting sites, as is potentially the case with *C. indicum* in this study. In a previous investigation on the development of an attractive toxic sugar bait to target *An. arabiensis*, Tenywa et al (Tenywa et al., 2017) discovered that the most efficient bait prototype drew mosquitoes in mostly for resting purposes, rather than feeding only. Unfortunately, we did not perform anthrone tests on mosquitoes retrieved from odor traps during the multiple‐choice assays to determine the proportion of fructose‐positive mosquitoes. This would have helped distinguish whether plants were attractive for resting sites rather than for food sources, although the two might be linked.

Fourth, we did not characterize the full range of mosquito fitness‐related traits. The phenotypic traits measured here, that is, survival, insemination rate, and sugar intake, are not the only parameters determining mosquito fitness. For instance, it is possible that while *C. indicum* provided low survival and insemination rate, it may provide other important nutrients, including vitamins, amino acids, and secondary compounds, which could improve other parameters of mosquito fitness, such as fecundity, or defense against pathogens. Research on various animals has revealed that different life‐history traits can have distinct nutritional optima (Hawley et al., 2016 and references therein). For example, in the flesh fly *Sarcophaga crassipalpis*, lifespan was optimized at a lower total carbohydrate concentration compared to that for egg production (Hawley et al., 2016). Moreover, there is mounting evidence indicating that insects can use less nutritious food sources for purposes of self‐medication (de Roode et al., 2013). For instance, although the highly attractive *P. hysterophorus* (Manda, Gouagna, Nyandat, et al., 2007; Nikbakhtzadeh et al., 2014) appears to be a poor food source (Manda, Gouagna, Foster, et al., 2007; Nikbakhtzadeh et al., 2016), it contains parthenin (Nyasembe, Cheseto, et al., 2015), a toxin which has been shown to limit malaria parasite development within the mosquito (Balaich et al., 2016). In this regard, it is particularly relevant to note that extracts from flowers of *C. indicum* have strong antimicrobial properties (Kiruthika et al., 2011; Wessapan et al., 2007). Further studies should investigate the effect of this plant on a wider range of phenotypic traits, including mosquito egg production or defense against pathogens.

The design used for the performance experiments did not afford the mosquitoes the ability to select from multiple options as in a cafeteria design. We may expect different performance outcomes when mosquitoes would consume different proportions of each plant. In natural conditions, mosquitoes may visit *C. indicum* to acquire specific non‐sugar nutrients, and subsequently forage on another plant species to obtain sugars. Furthermore, the nutritional needs of insects are dynamic, undergoing constant changes, influenced by factors such as age and physiological status, as well as temperature and other environmental variables (Simpson et al., 2015). When considering these various pieces of information collectively, it becomes apparent that the plant preference pattern of mosquitoes, as observed in our laboratory setting, may not only diverge but also serve as an indication that the preference exhibited for *C. indicum* is not necessarily maladaptive in natural environments.

The effects of the various treatments (*I. coccinea*, *C. pulcherrima*, *C. indicum*, and the 5% glucose solution) on mosquito survival were not consistent across our three tests (Experiments 1.1, 1.2, and 1.3). For instance, in the first and third tests, *I. coccinea* provided similar survival rates to that of the 5% glucose solution, while in the second test, survival on *I. coccinea* was lower compared to that achieved with the 5% glucose solution. Similarly, while in Experiments 1.1 and 1.3 (as well as in an additional survival experiment presented in Figure S13), the survival of mosquitoes fed with *C. indicum* was similar to that of mosquitoes fed with water only, *C. indicum* provided much better survival than water in Experiment 1.2. It is important to note that these experiments were conducted at different time periods using distinct groups of mosquitoes and plant individuals/populations, leading to variability in both insect and plant materials. Furthermore, we utilized plants collected from the field, which were naturally exposed to a whole community of nectar‐feeding animals. Therefore, it is plausible that the nectar abundance could have fluctuated depending on the collection period (phenology of the plant) or the identity of the flowers collected, leading to variation in plant‐mediated effects on mosquito survival. This perspective offers another possibility for elucidating the mismatch between performance and preference observed in our experiments: in natural settings, *C. indicum* flowers might be highly attractive (as observed in our multiple‐choice laboratory assays) and entice a multitude of consumers (including wild mosquitoes), depleting its nectar and leaving our mosquitoes with nothing during the no‐choice experiments. A suggestion for future studies is to use mesh bags to protect the flowers from nectar depletion by other nectar‐feeding animals.

Among possible nectar consumers, there is a growing awareness about the importance of nectar microbes in insect–plant interactions (Herrera et al., 2008; Vannette, 2020). These microorganisms can not only alter the sugar content of nectar but they can also influence the attraction of floral visitors, including mosquitoes (Peach et al., 2021). Tri‐trophic relationships involving plants, microbes, and mosquitoes may introduce additional layers of complexity to the observed outcomes. In particular, the microbial community associated with nectar of *C. indicum* could produce olfactory cues that affect mosquito attraction, potentially altering the perceived reward associated with this plant species. Future research exploring the microbial dynamics within floral nectar and their impact on mosquito behavior could provide further insights into the multifaceted nature of these relationships.

Our behavioral assay unveiled a heightened responsiveness of *An. gambiae* toward plants in comparison to *An. coluzzii*, leading to a greater activation rate. The underlying reasons remain ambiguous, but intrinsic genetic factors may play a role. Prior research conducted on male swarms of *An. coluzzii* from the Vallée du Kou and *An. gambiae* from Soumousso (the two locations from which our mosquito colonies were derived) demonstrated that swarming *An. gambiae* males possessed a higher total sugar reserve compared to their *An. coluzzii* counterparts (Maïga et al., 2014). This observation suggests a potentially innate predisposition for greater sugar‐feeding tendencies in *An. gambiae* relative to *An. coluzzii*, although ecological differences between the two sites may also account for this discrepancy and our cold‐anthrone test suggested equivalent levels of sugar positivity between the two species. In the performance experiments, there were also some differences in the responses of *An. coluzzii* and *An. gambiae* to the plant treatment, but these were more nuanced than in the preference experiment. Overall, our intention was to establish a foundation for future investigations that can delve deeper into potential interspecific differences, rather than drawing definitive species‐specific conclusions.

In previous studies, *Anopheles* preference for plants has been assessed using either a dual‐port Y olfactometer (Gouagna et al., 2014; Nikbakhtzadeh et al., 2014) or by direct observations of mosquitoes perching or feeding on plants (Manda, Gouagna, Nyandat, et al., 2007). The novel multiple‐choice test device employed in this study, inspired by the odor‐baited net traps utilized to measure mosquito preference for vertebrate hosts (Tangena et al., 2015; Vantaux et al., 2021), demonstrated remarkable reliability in assessing the behavioral response of mosquitoes toward different plant species. Notably, the two control traps baited with either water or 5% glucose solution, which do not emit volatiles, yielded very few mosquito captures. This successful validation of the device eliminates the need for human observation or video recording techniques and enables comparisons across a larger number of plants.

The cutting of flowers, as employed in our experiments and in previous studies, introduces the potential risk of stress‐related phytochemical release, which might influence the behavioral responses of mosquitoes. While various studies have demonstrated the inducible production of chemical defense compounds by leaves following herbivory or mechanical damage (War et al., 2012), the exploration of such emissions from flowers is limited. Notably, a study by Kishimoto & Shibuya (Kishimoto & Shibuya, 2021) directly compared scent emissions between intact and cut flowers. Despite finding no significant difference in volatile composition or abundance between cut and intact flowers, the temporal dynamics varied, with total olfactory emissions maintained for approximately 6 days in intact flowers versus 2 days in cut flowers. In the context of our study, where flowers were cut between 3 and 4 p.m. and offered to mosquitoes at 5 p.m. throughout the night, it is reasonable to assume that odors were still being emitted when the flowers were exposed to mosquitoes, although plant species‐specific patterns cannot be excluded. Ideally, future studies should explore comparisons between cut experimentally grown potted flowers and uncut counterparts to directly assess the impact of damage‐induced stress volatiles on mosquito behavioral responses. However, the majority of the 31 plant species selected for our screening experiment are sourced from trees or shrubs, making the use of potted flowers challenging.

While our study has provided insights into the performance and preference of *An. coluzzii* and *An. gambiae* mosquitoes toward different plant species, definitive confirmation of these interactions requires complementary approaches, such as amplifying and sequencing genetic markers of plant DNA residues found in field‐collected mosquito crops and midguts. Recent studies employing this approach have identified some plant families used by *Aedes* and *Anopheles* mosquitoes (Junnila et al., 2010; Nyasembe et al., 2018; Upshur et al., 2023; Wanjiku et al., 2021). Applying eDNA barcoding will offer validation of the actual utilization of plant species identified in our study as nutritional sources for *An. coluzzii* and *An. gambiae* in their natural habitat in West Africa.

## 5 CONCLUSIONS

*Anopheles gambiae* and *An. coluzzii* encounter diverse plant communities in their environment, providing them an opportunity to feed selectively on a range of plant species that can play crucial roles in their life history. Consistent with prior research, our findings revealed that different plant species elicited varying levels of mosquito survival in no‐choice assays. However, contrary to our initial hypothesis, which posited that mosquitoes would exhibit a preference for certain species based on perceived differences in resource quality, we observed highest preference for the plant species that resulted in the lowest survival rates. This intriguing finding suggests the existence of alternative mechanisms influencing *Anopheles* preference for plants. The exploration of mosquito–plant relationships has long been overlooked, but there is immense potential for future research to build upon the foundational knowledge established in other insect systems, such as *Drosophila* flies, *Spodoptera* moth caterpillars, or locusts. The exploration of the aforementioned questions (e.g., do mosquitoes solely seek food (sugar) when selecting plants?; how do plants influence a range of specific mosquito fitness traits in varying ways?; and how does plant quality vary across species/individuals and changing environments?) can greatly benefit from adopting a nutritional ecology framework (Raubenheimer et al., 2009). This approach may provide a comprehensive understanding of the meaning of plant quality for mosquito performance and the extent to which mosquitoes are able to actively regulate their nutrition through plasticity in behavioral preference. Enhancing our comprehension of mosquito–plant interactions can also be pivotal for the improvement of attractive toxic sugar bait strategies and for the advancement of synthetic plant odor lures used in malaria vector control efforts in Africa.

## AUTHOR CONTRIBUTIONS

**Prisca S. L. Paré:** Conceptualization (equal); formal analysis (equal); methodology (equal); writing – original draft (equal); writing – review and editing (equal). **Domonbabele F. D. S. Hien:** Conceptualization (equal); funding acquisition (equal); investigation (equal); methodology (equal); supervision (equal); writing – review and editing (equal). **Mariam Youba:** Methodology (equal); writing – review and editing (equal). **Rakiswendé S. Yerbanga:** Writing – review and editing (equal). **Anna Cohuet:** Writing – review and editing (equal). **Louis‐Clément Gouagna:** Writing – review and editing (equal). **Abdoulaye Diabaté:** Writing – review and editing (equal). **Rickard Ignell:** Writing – review and editing (equal). **Roch K. Dabiré:** Writing – review and editing (equal). **Olivier Gnankiné:** Conceptualization (equal); supervision (equal); writing – original draft (equal); writing – review and editing (equal). **Thierry Lefèvre:** Conceptualization (equal); formal analysis (equal); funding acquisition (equal); investigation (equal); supervision (equal); writing – original draft (equal); writing – review and editing (equal).

## FUNDING INFORMATION

This work was supported by the Rakiswendé IRD JEAI Grant No. AAP2018_JEAI_PALUNEC and ANR Grant STORM No.16‐CE35‐0007.

## CONFLICT OF INTEREST STATEMENT

The authors declare no competing interests.

## Supporting information


Figures S1–S14.

Tables S1–S16.

## Data Availability

All data and R code files are available at the following link: https://dataverse.ird.fr/dataset.xhtml?persistentId=doi:10.23708/JF9SCW.
